# New Ultrasensitive Sandwich-Type Immunoassay of Dendritic Tri-Fan Blade-like PdAuCu Nanoparticles/Amine-Functionalized Graphene Oxide for Label-Free Detection of Carcinoembryonic Antigen

**DOI:** 10.3390/mi12101256

**Published:** 2021-10-16

**Authors:** Pingping Xu, Wenpo Feng, Mei Wang, Ling Zhang, Gaofeng Liang, Aihua Jing

**Affiliations:** 1 School of Medical Technology and Engineering, Henan University of Science and Technology, Luoyang 471023, China; 190319221055@stu.haust.edu.cn (P.X.); 200320221489@stu.haust.edu.cn (M.W.); zhangling019@haust.edu.cn (L.Z.); 2 Medical College, Pingdingshan University, Pingdingshan 467000, China; fwp238@haust.edu.cn; 3 Medical College, Henan University of Science and Technology, Luoyang 471023, China; lgfeng990448@haust.edu.cn

**Keywords:** dendritic PdAuCu nanoparticles, amine, graphene oxide, signal amplification, sandwich-type electrochemical immunosensor, carcinoembryonic antigen

## Abstract

The early detection of tumor markers has an effective role in the treatment of cancer. Here, a new sandwich-type electrochemical immunosensor for early label-free detection of the cancer biomarker carcinoembryonic antigen (CEA) was developed. Dendritic tri-fan blade-like PdAuCu nanoparticles (PdAuCu NPs)/amine functionalized graphene oxide (NH_2_-GO) were the label of secondary antibodies (Ab_2_), and Au nanoparticle-decorated polydopamines (Au/PDA) were immobilized on a screen-printed carbon electrode (SPCE) as the substrate materials. Dendritic tri-fan blade-like PdAuCu NPs/NH_2_-GO was synthesized according to a simple hydrothermal procedure and used to immobilize antibodies (Ab_2_) with large surfaces areas, increased catalytic properties and good adsorption to amplify the current signals. Subsequently, Ab_2_/PdAuCu NPs/NH_2_-GO catalyzed the reduction of H_2_O_2_ in the sandwich-type immunoreactions. Under optimal conditions, the immunosensor exhibited a satisfactory response to CEA with a limit detection of 0.07 pg mL^−1^ and a linear detection range from 0.1 pg mL^−1^ to 200 ng mL^−1^. The proposed immunosensor could be suitable enough for a real sample analysis of CEA, and has clinical value in the early diagnosis of cancer.

## 1. Introduction

Malignant tumors are characterized by high morbidity and mortality and a serious dangers to human health [1,2]. Carcinoembryonic antigen (CEA), a broadly studied tumor marker, is an attractive clinical cancer indicator in colon tumors, breast tumors, ovarian carcinomas, colorectal cancer and cystadenocarcinomas [3]. The sensitive and accurate detection of tumor markers in serum is vital for early diagnoses and clinical treatment of tumors [4]. To date, various sophisticated methods have been used to determine biomarkers, including immunohistochemistry (IHC) [5], real-time PCR [6], microarray chips [7], fluorescence in situ hybridization (FISH) [8,9], chromogenics [10], chemiluminescence immunoassay [11,12] and electrochemistry [13]. Among these strategies, as we know, high specificity is the reason why enzyme-linked immunosorbent assay (ELISA) has been used as the conventional method for the detection of cancer biomarkers. Therefore, immunosensors rely on the specificity of an antibody to its corresponding antigen, which has attracted focus in the assay of CEA. Optical and electrochemical immunosensors are two of the most-used methods to detect CEA with high sensitivity. Optical immunosensors, for example, fluorometric immunosensors, need a fluorescent dye and fluorescence quenching probe. Photoelectrochemical (PEC) immunosensors can convert photoirradiation to an electrical signal in an assay for CEA analysis. Electrochemical immunosensors, based on the interaction of an antigen and its corresponding antibody, have attracted considerable attention in the fields of food safety control, environmental pollutant monitoring, clinical analysis and the diagnosis of cancers due to their distinct advantages, such as high selectivity, simple instrumentation, excellent sensitivity and low detection limits. Specifically, electrochemical immunosensors based on nanomaterials with high selectivity and excellent sensitivity to quantify biomarkers in clinical diagnostics are outstanding methods. Antibodies are adsorbed on the surface of the immunosensor to capture specific biomarkers by antibody–antigen interactions. The interface of the immunosensors with high immobilizing efficiency is vital for signal amplification and therefore increasing the immunosensor’s sensitivity. As a result, effective nanomaterials are in urgent demand.

Graphene, a two-dimensional material with a honeycomb crystal lattice, has attracted considerable interest due to its multiple fascinating electronic, optical and thermal properties [14]. Graphene is a potential material in the fabrication of electrochemical immunosensors due to its unique nanostructure and excellent conductivity. Our group has developed 3D Au/holey-graphene [15] and 3D porous nanoplatinum/graphene [16] electrochemical sensors. Gao Yansha’s team developed a novel immunosensor based on Nile blue A-reduced graphene oxide for the detection of the carcinoembryonic antigen [17]. Gold (Au)-based tri-metallic nanomaterials have good electrical conductivity and outstanding biocompatibility and have been used in immunosensors to capture antibodies [18,19]. Dendritic nanoparticles (NPs) on the interface of an electrode can provide a superior microenvironment for anchoring a greater number of antibodies and facilitate electron transfer due to more active sites on their surface, and therefore enhance the sensitivity of the biosensor. Thus, in this research, dendritic tri-fan blade-like PdAuCu nanoparticle (PdAuCu NPs)-decorated amine-functioned graphene oxide was selected as the matrix material to assemble an immunosensor for the selective and reproducible electrochemical detection of cancer biomarkers.

**Figure 1:**
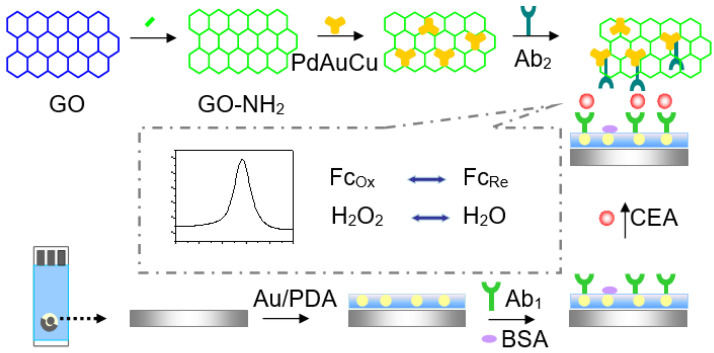
The schematic illustration of the fabrication procedure of the CEA immunosensor.

A screen-printed carbon electrode (SPCE) is a kind of biosensing tool made by screen-printing technology. Such tools are portable for on-site and point-of-care detection and are used for their unique characteristics, such as their easy miniaturization, lower cost, disposable nature, and mass production. They monitor physiological indicators in the process of diagnosis. In this work, an SPCE was employed to fabricate an electrochemical immunosensor for the ultrasensitive detection of CEA (Figure 1). First, a modified SPCE was fabricated to adsorb the antibody (Ab_1_) owing to its advantages such as simplicity, low cost, small size, rapid responses, and easy mass production [20,21]. Au NPs-functionalized polydopamine (Au/PDA) was deposited on the surface of SPCE to increase the performance of the immunosensor. This is due to the large number of amino groups of PDA molecules that can capture plenty of the primary antibody through covalent bonds. Moreover, dendritic tri-fan blade-like PdAuCu NPs were synthesized and decorated with amine group-functioned graphene oxide (NH_2_-GO). After that, dendritic tri-fan blade-like PdAuCu NPs/NH_2_-GO was selected as the matrix material to capture Ab_2_. The specific nanostructures and the multi-metals’ electronics enhance the high sensitivity of the immunosensor. The signal of the proposed immunosensor was linear from 0.1 pg mL^−1^ to 200 ng mL^−1^ CEA and had a low detection limit of 0.07 pg mL^−1^ (S/N = 3). The developed immunosensor was tested in CEA detection in human serum samples and urine samples, which can potentially be a bridge to a miniaturized portable potentiostat working with microvolumes and for point-of-care analysis.

## 2. Materials and Methods

### 2.1. Materials and Equiments

Graphite powder was prepared in our laboratory. CEA, Palladous chloride (PdCl_2_) and chloroauric acid (HAuCl_4_) were purchased from Sangon Biotech (Shanghai, China). The mouse-derived McAb to CEA was purchased from Linc-Bio Science Co. Ltd. (Shanghai, China). HRP-conjugated rabbit anti-mouse IgG was purchased from Sangon Biotech (Shanghai, China). Bovine serum albumin (BSA) was obtained from Beijing Cell Chip Biotechnology Co., Ltd. (Beijing, China). Ethylene diamine was obtained from Tianjin Yongda chemical reagent company, 1-(3-Dimethylaminopropyl)-3-ethylcarbodiimide hydrochloride (EDC) and N-Hydroxysuccinimide (NHS) were obtained from Tianjin BASF chemical company (Tianjin, China). Sodium cyanoborohydride (NaCNBH_3_) and ferrocene carboxaldehyde (Fc-CHO) were purchased from Macklin (Shanghai, China). Polyvidone (PVP), L-ascorbic acid (AA), sodium citrate, cetrimonium bromide (CTAB), sodium borohydride (NaBH_4_) and dopamine (DA) were purchased from Aladdin Chemistry Co. Ltd. (Shanghai, China). The chemicals and solvents used in the experiments were of analytical grade. Milli-Q water was used throughout the experiments. Phosphate-buffered saline (PBS, 10 mmol/L, pH 7.4) was prepared by dissolving 8 g NaCl, 0.2 g KCl, 2.9 g Na_2_HPO_4_·12H_2_O and 0.24 g KH_2_PO_4_ in 1 L of distilled water. Blocking buffer was prepared by dissolving 3.0% (w/v) BSA in PBS.

Transmission electron microscopy (TEM) images were obtained by a JEOL 2010 transmission electron microscope and an FEI Talos F200× transmission electron microscope. The electrochemical detection was performed on disposable SPCE with a 3 mm diameter working area (Zensor research and development, Taiwan), connected to a CHI 660E workstation (Chenhua, Shanghai, China).

### 2.2. Preparation of NH_2_-GO

GO was synthesized according to a previously reported procedure [22]. GO powder (0.5 g) was added to 100 mL DMF solution and ultrasonicated for 30 min. EDC (1.0 g) and NHS (1.0 g) were then added into the above solution and stirred for 3 h. An amount of 1.35 g ethylene diamine was then added into the above solution and stirred for 12 h at room temperature. The solution was centrifuged and washed several times with DMF. After dialysis for 1 day, the desired product, NH_2_-GO, was obtained.

### 2.3. Preparation of Dendritic Tri-Fan Blade-Like PdAuCu NPs

Dendritic tri-fan blade-like PdAuCu NPs was synthesized by the method described previously [19,23]. Firstly, 2.5 mL of PdCl_2_ (20 mmol L^−1^), 0.8 mL of HAuCl_4_ (24 mmol L^−1^), 1.0 mL of CuCl_2_ (20 mmol L^−1^), 200 mg of KBr and 0.2 mL of HCl solution (6 mol L^−1^) were sequentially put into 10 mL of a PVP solution (0.01%) under constant stirring. Then, 2.0 mL of L-ascorbic acid (AA) solution with a concentration of 0.1 mol L^−1^ was put into the mixed solution and then reacted in an oil bath for 30 min at 95 °C. Finally, the resulting product was efficiently washed with ethanol and centrifuged at 6000 rpm, followed by drying in a vacuum oven at 60 °C.

### 2.4. Preparation of Fc-NH_2_-GO

Fc-NH_2_-GO was synthesized according to the literature with a little modification [24]. A volume of 10 mL of the prepared AG solution (5.0 mg mL^−1^) and 10 mL of Fc-CHO methanol solution (3.0 mg mL^−1^) were mixed under stirring at 25 °C for 2 h. Subsequently, 100 mg of sodium cyanoborohydride (NaCNBH_3_) was put into the mixed solution and reacted for 24 h at room temperature. After a freshly prepared NaOH solution (5%) was added in the mixture, the product was washed with pure water and centrifuged at 6000 rpm for 5 min.

### 2.5. Preparation of Au/PDA Nanoparticles

Firstly, 0.6 mL 1% HAuCl_4_ and 0.2 mL K_2_CO_3_ were added into 40 mL ice-cold pure water with stirring. Then, 0.4 mL 1.0 mg mL^−1^ of NaBH_4_ was quickly added in five times until the solution turned orange-red. The mixture was continuously stirring for 5 min. A total of 10 mL of the above-obtained Au NPs solution was added into 10 mL of Tris-HCl buffer (10 mM, pH = 8.5). After adding 20 mg of DA, the solution was stirred at room temperature for 24 h. The Au/PDA product was centrifuged and washed with ultrapure water several times and resuspended with pure water [25].

### 2.6. Preparation of Ab_2_/HRP-Dendritic PdAuCu NPs/Fc-NH_2_-GO Bioconjugate

Figure 1 illuminates the preparation procedures of Ab_2_/HRP-dendritic PdAuCu NPs/Fc-NH_2_-GO bioconjugate. Specifically, 8 mg of NH_2_-GO was dissolved into 2 mL of a phosphate-buffered solution (PBS, 0.10 mol L^−1^, pH 7.4) and ultrasonicated for 20 min, followed by adding 2.0 mL of the dendritic tri-fan blade-like PdAuCu suspension (4 mg mL^−1^) under stirring for 30 min. Next, the above mixture (1.0 mL) and Ab_2_ (20 μg mL^−1^, 2.0 mL) were well mixed and stirred for 12 h at 4 °C. Subsequently, 0.1% BSA (60 μL) was added to completely hinder the inactive sites on the surface of dendritic tri-fan blade-like PdAuCu NPs/Fc-NH_2_-GO. Finally, the resulting Ab_2_/HRP-dendritic PdAuCu NPs/Fc-NH_2_-GO bioconjugate was centrifuged, re-dispersed in the PBS (pH = 7.0) and stored at 4 °C for further use.

### 2.7. Fabrication of the Electrochemical Immunosensor

Figure 1 shows a schematic diagram of the fabrication procedure of the immunosensor. A total of 6.0 μL of the Au/PDA (2.0 μg mL^−1^) was dropped onto the active areas (0.3 cm in diameter) of the SPCE and dried in air; then, 6 μL of anti-CEA (20 μg mL^−1^) was dropped evenly onto the prepared SPCE surface. The electrode was cleaned with pH 7.4 PBS and incubated at 4 °C for 12 h and then was incubated in 1% BSA solution for an hour at 37 °C to block the remaining active sites to avoid non-specific adsorption. The immunosensor was further cleaned with PBS and dried with a high-purity nitrogen steam. Subsequently, the sensor was incubated in CEA solutions at various concentrations for 30 min at 37 °C, washed with PBS solution and dried under a steam of nitrogen. Finally, the immunosensor was immersed in Ab_2_/HRP-PdAuCu NPs/Fc-NH_2_-GO bioconjugate solution prepared in Section 2.6 for 30 min for sandwich immunoreaction. After cleaning with 0.01 M PBS, the immunosensor was dried using nitrogen gas.

### 2.8. Electrochemical Measurements

The electrochemical measurements were performed on 50 μL of a solution containing 5 mM K_3_[Fe(CN)_6_] and 5 mM K_4_[Fe(CN)_6_] prepared in PBS with pH 7.4. Cyclic voltammograms (CV), differential pulse voltammetry (DPV) and electrochemical impedance spectroscopy (EIS) were performed in the electrolyte solution (10 mM PBS (pH 7.4) + 2 mM [Fe(CN)_6_]^3−^/[Fe(CN)_6_]^4−^(1:1) + 0.1 M KCl).

## 3. Results and Discussion

### 3.1. Characterization of PdAuCu NPs

**Figure 2:**
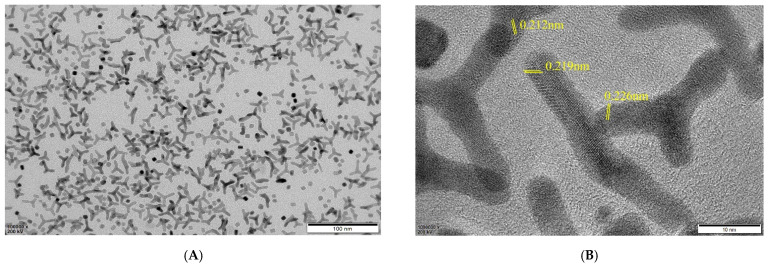
TEM images of PdAuCu NPs at low (**A**) and high magnification (**B**).

Figure 2 shows TEM images of the typical PdAuCu NPs at different magnifications.

The low magnification images displayed lots of dendritic structures, just as the tri-fan blades (Figure 2A), which are well-dispersed with a length of more than 20 nm and a width of 5 nm. Meanwhile, the high-resolution TEM (HRTEM) image (Figure 2B) illuminates a large number of clearly visible lattice fringes. Impressively, there is a high density of atomic steps displayed on the edges of crystal surfaces. The anisotropic structure, which would provide more catalytic active sites, accordingly effectively enhances the electron transfer rates. Moreover, a series of lattice fringes with d-spacing values of 0.212 nm and 0.219 nm were shown, just between that of Cu (0.208 nm) and Pd (0.224 nm), which corresponded to the (111) plane of the PdCu alloy. In addition, a d-spacing of 0.226 nm was also observed, owing to the incorporation of Cu and Au into the Pd lattice, which indicated a more forward fabrication of the PdAuCu nanocrystals [23,26,27].

**Figure 3:**
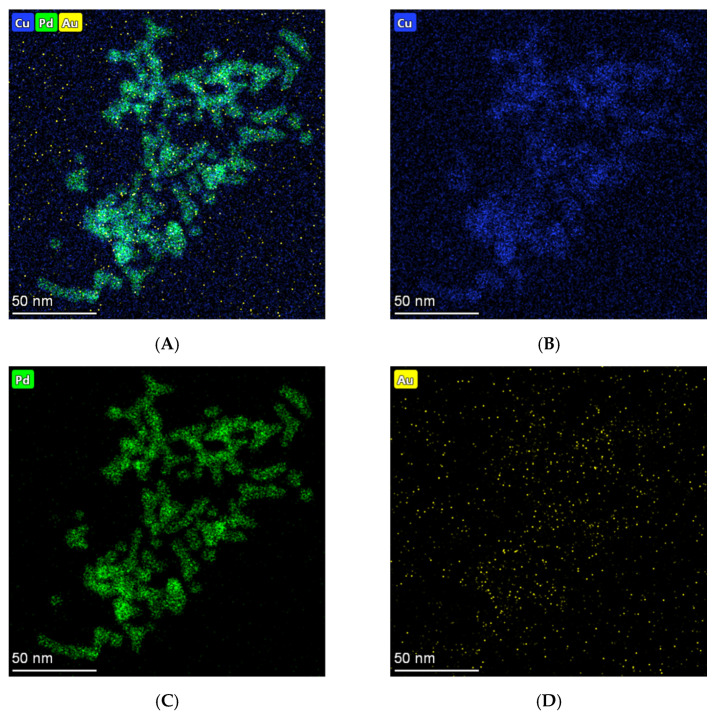
The elemental mapping of (**A**) the overlay of PdAuCu NPs, (**B**) Cu, (**C**) Pd, and (**D**) Au.

According to the elemental mappings (Figure 3A–D), Pd (green signal), Au (yellow signal) and Cu (blue signal) atoms distributed throughout the entire structures further revealed the ternary constitute of PdAuCu NPs. Among them, most Pd atoms emerge on the branches of the nanoparticles, which would improve the catalysis. Obviously, the above characterization demonstrated successful formation of the PdAuCu NPs with tri-fan blade-like structures ternary alloy compound.

### 3.2. Electrochemical Characteristics of the Immunosensor

**Figure 4:**
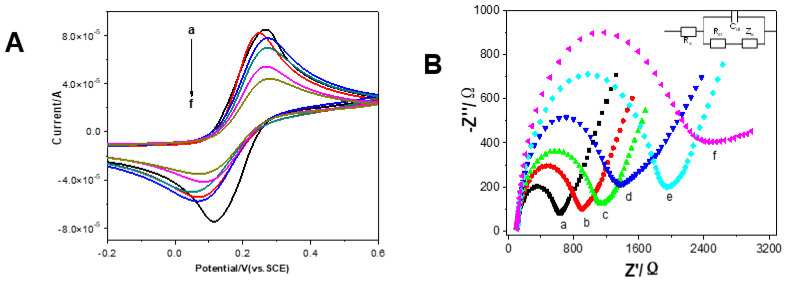
(**A**) CVs and (**B**) Nyquist plots of bare SPCE (a), Au/PDA (b), Ab_1_/Au/PDA (c), BSA/Ab_1_/Au/PDA (d), CEA/BSA/Ab_1_/Au/PDA (e), and Ab_2_/CEA/BSA/Ab_1_/Au/PDA (f), modified SPCE in 0.10 M KCl containing 2 × 10^–3^ M K_3_[Fe(CN)_6_]/K_4_[Fe(CN)_6_].

CV and EIS are common methods to investigate the fabrication process of the electrochemical immunosensor. Figure 4A shows the CVs of Fe(CN)_6_^3−^/Fe(CN)_6_^4−^ in PBS at the bare SPCE (curve a), Au/PDA/SPCE (curve b), Ab_1_/Au/PDA/SPCE (curve c), BSA/Ab_1_/Au/PDA/SPCE (curve d), CEA/BSA/Ab_1_/Au/PDA/SPCE (curve e) and Ab_2_/CEA/BSA/Ab_1_/Au/PDA/SPCE (curve f), respectively. The changes in the amperometric response correspond to the obstruction of the electron transfer kinetics of the Fe(CN)_6_^3−^/Fe(CN)_6_^4−^ probe. It can be seen from Figure 4A that the anodic and cathodic current of Au/PDA/SPCE was higher than that of the bare SPCE, which means that Au/PDA/SPCE showed better electron transfer performance than bare SPCE. This was as a result of the excellent conductivity material that Au/PDA modified on SPCE, facilitating the electron transfer. After Ab_1_, BSA, CEA, and Ab_2_ were sequentially assembled on the surface of the electrodes, the peak current decreased accordingly. This is because the blocking layer of the molecules of Ab_1_, BSA, CEA, and Ab_2_ modified on the electrode surface hindered the electron transfer between the probe and the electrode surface [28].

The EIS measurement is another way monitoring the modification steps on the interface of SPCE in the [Fe(CN)_6_]^3−^/[Fe(CN)_6_]^4−^ probe system. The impedance spectrum includes a semicircular fragment and a linear fragment. The semicircle part is associated with the electrochemical process of electron transfer process, and the linear part corresponds with the diffusion process [29,30,31,32]. The electron transfer resistance (R_et_) is associated with the diameter of the semicircle fragment. As shown in Figure 4B, the Au/PDA-modified SPCE (curve b) displayed a smaller semicircle than the bare SPCE (curve a) at high frequencies, implying that it has lower Ret values than SPCE. Meanwhile, that Au/PDA/SPCE possesses good conductivity and can improve electron transfer effectively. Subsequently, after Ab_1_ (c), BSA (d), CEA (e), and Ab_2_ (f) were assembled on the electrode step by step, and the Ret value grew larger gradually. This was because of the molecules on SPCE blocking the electron transfer on the [Fe(CN)_6_]^3−^/[Fe(CN)_6_]^4−^ probe. These EIS and CV results indicated that the immunosensor was successfully fabricated. The results were consistent with previous studies [23,32]. Inset in Figure 4B, the Randles equivalent circuit is illustrated. (R_s_: the solution resistance. R_ct_: the charge transfer resistance. C_dl_: the double layer capacitance. Z_w_: the Warburg impedance.)

The electrochemical characterization of Au/PDA/SPCE was investigated in PBS solution (10 m mol L^−1^ PBS (pH 7.4) + 0.1 mol L^−1^ KCl + 2 mM of K_3_[Fe(CN)_6_] + 2 mM K_4_[Fe(CN)_6_]) at different scan rates (10~400 mV s^−1^). It was found that the redox peak current increased linearly with the square root of the scan rate. This indicated that the reaction occurring on the surface of Au/PDA/SPCE is a diffusion-controlled process, according to the Randles–Sevcik equation: [28,33].

I_p_ = (2.69 × 10^5^) n^3/2^ ACD^1/2^υ^1/2^

where I_p_ (A) is the anodic peak current, n is the number of electron transfers, A (cm^2^) is the surface area of the electrode, C (mol cm^−3^) is the concentration of K_3_[Fe(CN)_6_], D (cm^2^ s^−1^) is the diffusion coefficient and υ (V s^−1^) is the scan rate.

### 3.3. Optimization of Synthesis Conditions of Nanocomposites and Immunoassay Conditions

**Figure 5:**
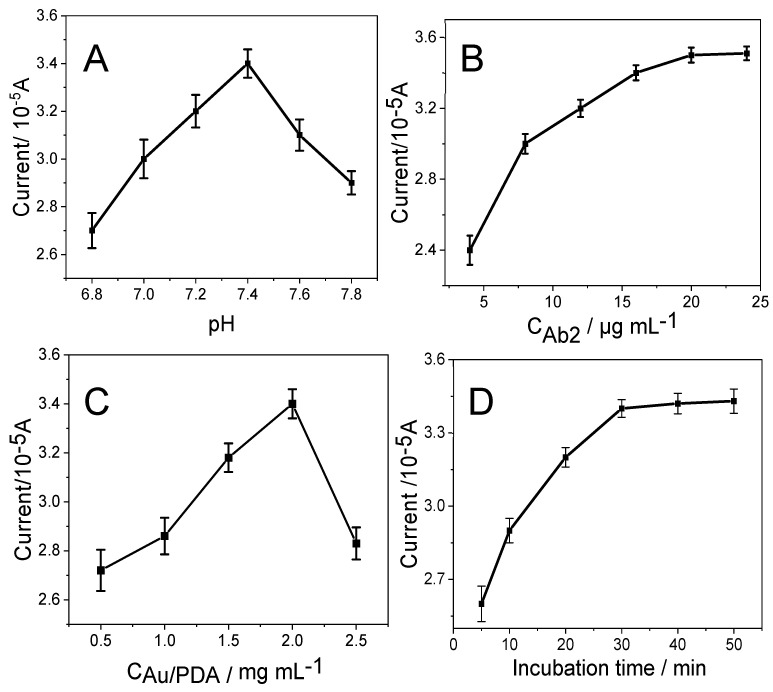
Effect of (**A**) pH value, (**B**) the Ab_2_/HRP-dendritic PdAuCu NPs/Fc-NH_2_-GO concentration, (**C**) Au/PDA concentration and (**D**) the incubation time on the DPV response during the detection of CEA. Error bar = RSD (*n* = 5).

To achieve the optimal performance of the immunoassay, the solution pH value, the amount of immobilized Ab_2_, the concentration of Au/PDA and the incubation time were tested. The influences of solution pH on the responses to detect CEA are illustrated in Figure 5A. The current increased when pH values were changed from 6.8 to 7.4; after that, it decreased from pH 7.4. Thus, PBS at pH 7.4 was selected for further detection.

In addition, the optimal concentration of Ab_2_/HRP-Dendritic PdAuCu NPs/Fc-NH_2_-GO on the immunosensor has been investigated. The peak current increased when the concentration of Ab_2_/HRP-dendritic PdAuCu NPs/Fc-NH_2_-GO increased from 5 μg mL^−1^ to 20 μg mL^−1^, and remained constant over 20 μg mL^−1^ (Figure 5B). This is perhaps a lower concentration of Ab_2_/HRP-dendritic PdAuCu NPs/Fc-NH_2_-GO resulting in not enough Ab_2_ to capture CEA. Additionally, a 20 μg mL^−1^ concentration of Ab_2_/HRP-dendritic PdAuCu NPs/Fc-NH_2_-GO captures CEA at the point of saturation on the surface of SPCE. Therefore, 6 μL of 20 μg mL^−1^ of Ab_2_/HRP-dendritic PdAuCu NPs/Fc-NH_2_-GO was selected for the electrochemical immunosensor [34].

Moreover, the concentration of Au/PDA is an important factor for the behavior of the immunosensor. As shown in Figure 5C, an increase in the concentration of Au/PDA was related to an increase in the current response of the immunosensor during the detection of CEA, implying that a higher load of Ab_1_ on Au/PDA results in a larger amount of antigen binding. Nevertheless, when the concentration of Au/PDA was greater than 2.0 mg mL^−1^, the peak currents decreased due to an increase in the film of Au/PDA thickness, which has an interface on the electron transfer. Additionally, SPCE-modified 2.0 mg mL^−1^ of Au/PDA has the best electrocatalytic response. Therefore, 2.0 mg mL^−1^ Au/PDA was chosen for the assembly of the immunosensors.

Figure 5D illustrates the relationship between the incubation time and the electrocatalytic response of the immunoassay. A longer incubation time acquired higher DPV responses for CEA. After 30 min, the DPV current tends to stay constant. This indicates that CEA antigen adsorption by the captured antibody reached saturation; meanwhile, the binding of the captured antibody to Ab_2_/HRP-dendritic PdAuCu NPs/Fc-NH_2_-GO on the SPCE surface also reached saturation. Therefore, a 30 min incubation time was selected as the optimal incubation time [18,35].

### 3.4. Quantitative Detection of CEA

**Figure 6:**
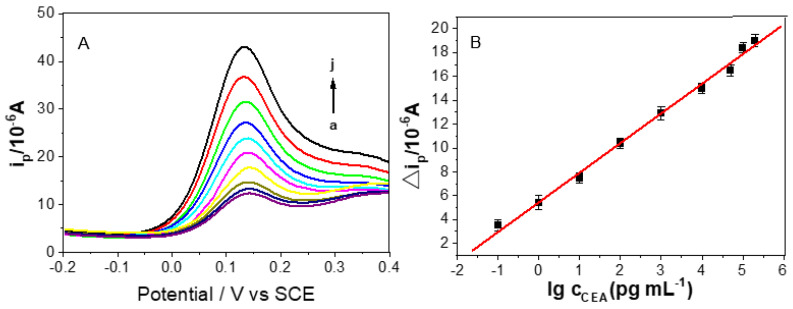
(**A**) DPV responses of the proposed immunosensor after incubation with various concentrations of CEA in 0.01 M PBS (pH 7.4) (a–j: 0, 0.1, 1, 10, 100, 1000, 10,000, 50,000, 100,000, 200,000 pg·mL^−1^). (**B**) Linear relationship between the ΔI of the immunosensor and logarithm of CEA concentration. Error bars represent the standard deviation, *n* = 5.

DPV has higher sensitivity than CV and is usually used for quantitative detection in electrochemistry. Under optimal conditions, the prepared sandwich-type immunosensor using Ab_2_/HRP-dendritic PdAuCu NPs/Fc-NH_2_-GO as labels and Au/PDA as substrate materials was used to detect CEA by DPV in 0.01 M PBS (pH 7.4) from −0.2 to 0.4 V. The current change was mainly attributed to the interaction between the labels of Ab_2_/HRP-dendritic PdAuCu NPs/Fc-NH_2_-GO and the substrate of Au/PDA, and the current toward CEA concentrations were displayed in Figure 6. It can be seen clearly that the signal intensity of the CEA detection of the proposed immunosensor increased proportionally with an increase in CEA concentration. These curves, from curve a to curve j in Figure 6A, represent the electrocatalytic current responses of the as-prepared immunosensor incubated with different concentration of CEA: 0, 0.1, 1, 10, 100, 1000, 10,000, 50,000, 100,000 and 200,000 pg·mL^−1^. Figure 6B shows a linear relationship related to the logarithmic values of CEA concentration from 0.1 pg mL^−1^ to 200 ng mL^−1^, with a low detection limit of 0.07 pg mL^−1^ (S/N = 3). The regression equation of the calibration curve is: ΔI = 5.449 + 2.486 log CCEA (pg mL^−1^), with correlation coefficient of 0.9969. The comparison of the linear range and detection limit between our immunosensor and some published studies was listed in Table 1. The designed immunosensor illustrated an excellent sensitivity and a good liner range. The results are ascribed to the outstanding electronic conductivity of the Au/PDA, which can successfully adsorb Ab_1_, and the superior catalytic properties of dendritic tri-fan blade-like PdAuCu NPs/Fc-NH_2_-GO nanocomposite can magnify the electrocatalytic response to enhance sensitivity.

### 3.5. Selectivity, Reproducibility, and Stability of the Immunosensor

**Figure 7:**
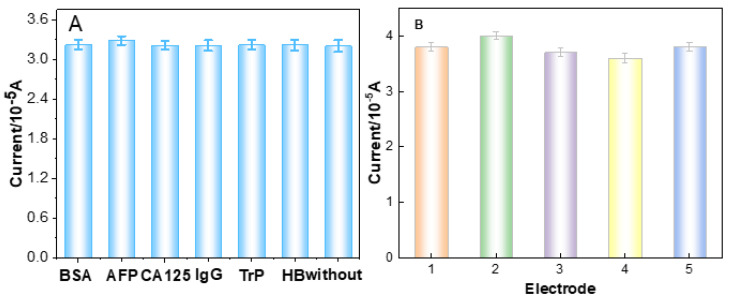
(**A**) Study of the specificity of the immunosensor towards various interferences: the currents towards 1.0 ng mL^−1^ CEA without and with 20.0 ng mL^−1^ BSA, AFP, CA125, IgG, Trp and Hb (error bar = SD, *n* = 3). (**B**) The reproducibility of the immunosensor.

The selectivity of the developed immunosensor is a significant parameter in the analysis of clinical samples. To confirm the selectivity of the proposed immunosensor to CEA, possible interference was determined by the same approach used in the case of CEA analysis; the test samples contained BSA, AFP, CA125, Trp, AFP, human IgG and hepatitis B surface antigen (HBs). The results are shown in Figure 7A. It found that the current signal variations of CEA containing interfering substances were less than 3.6% of those without interferences. The concentration of CEA was 1.0 ng·mL^−1^, while the concentrations of the other interferences were 20 ng·mL^−1^. The data indicate an excellent specificity towards CEA of the fabricated immunosensor.

The reproducibility of the immunosensor is another significant parameter in the quantitative CEA detection. The reproducibility of the immunosensor was investigated by detecting five parallel samples of CEA solutions (1.0 and 10.0 pg·mL^−1^) via DPV. The relative standard deviation of the five measurements was 3.14% and 2.48%, respectively, indicating that the proposed immunosensor has good reproducibility and satisfactory precision (Figure 7B).

Stability is a vital parameter to assess the performance of the immunosensor. The stability of the immunosensor was evaluated by detecting the changes of the DPV signals after the sensor was not used for a period of time. After storage at 4 °C for 7 days, the immunosensor retained 96.3% of its initial current peaks, indicating that the developed CEA immunosensor possessed an acceptable stability. Therefore, the developed immunosensor can be used for quantitative detection of CEA with high sensitivity, acceptable stability and reproducibility.

### 3.6. Real Sample Analysis

Based on the above research, the proposed immunosensor could be used to accurately analyze CEA in real samples. To demonstrate the application value of the immunosensor, various concentrations of CEA were added into human serum samples and urine samples by the standard addition method. The experimental results detected are displayed in Table 2. The recoveries ranged from 98% to 104.2% and the RSD ranged from 3.43% to 1.68%, indicating that the developed immunosensor is viable for accurate and quantitative detection of CEA in human serum for clinical diagnosis.

The high sensitivity, broader linear range and good stability of the immunosensor are largely attributed to the following factors: first, Au/PDA has excellent conductivity, attractive adsorption and a large surface area that ensures a greater number of Ab_1_ to sensitively detect trace CEA. Second, the hierarchical tri-fan blade-like PdAuCu NPs/Fc-NH_2_-GO structures, as well as synergistic effects of the tri-metals, provided more active sites to increase the catalytic activity. Third, the peroxidase-like catalytic activity of dendritic tri-fan blade-like PdAuCu NPs/Fc-NH_2_-GO has a high affinity with CEA that can enhance the sensitivity of the biosensor.

## 4. Conclusions

In summary, an ultrasensitive sandwich-type electrochemical immunoassay towards the trace detection of CEA was developed by Au/PDA and dendritic tri-fan blade-like PdAuCu NPs composites. Au/PDA with a large surface area and excellent conductivity was used as the substrate material of the immunosensor to capture Ab_1_. The dendritic tri-fan blade-like PdAuCu NPs/Fc-NH_2_-GO with superior peroxidase-like catalytic activity was used to capture Ab_2_/HRP. Under optimal conditions, a wider linear range of 0.1 pg·mL^−1^–200 ng·mL^−1^ and a low detection limit of 0.07 pg·mL^−1^ (S/N = 3) were observed. The developed immunosensor was applied to detect CEA in human serum samples and urine samples. The proposed electrochemical immunoassay has high selectivity, preeminent reproducibility and worthy stability, thus suggesting promising application prospects in disease treatment and clinical analysis.

## Figures and Tables

**Table 1:** Comparison of the characteristics of various modified electrodes.

Electrode	Detection Range (ng mL^−1^)	Detection Limit (ng mL^−1^)	Method	Refs.
NH_2_-G/THi/AuNPs	0.05–500	0.01	DPV	[36]
AuNP/IL/PICA/erGO	0.02–90	0.02	DPV	[37]
N, S-GQDs@Au-PANI	0.5–1000	0.01	CV	[38]
Fe_3_O_4_@Au NPs-S_1_-S_2_-S_3_	0.1–200	0.0004	CV	[39]
IL-rGO-AuNPs	0.01–100	0.01	DPV	[40]
PdAuCu NPs/Fc-NH_2_-GO	0.0001–200	0.00007	DPV	this work

**Table 2:** The results of CEA detection in the human serum samples and human urine samples.

Human Serum Samples					Human Urine Samples				
Samples	Added (ng mL^−1^)	Found (ng mL^−1^)	Recovery (%)	RSD (%)	Samples	Added (ng mL^−1^)	Found (ng mL^−1^)	Recovery (%)	RSD (%)
1	0.05	0.0512	102.4	3.02	7	0.05	0.0493	98.6	2.75
2	0.5	0.490	98.00	2.87	8	0.5	0.492	98.4	2.21
3	1.00	0.983	98.30	3.43	9	1.00	1.027	102.7	1.83
4	5.00	5.19	103.80	2.16	10	5.00	5.18	104.2	3.14
5	50.00	49.52	99.04	2.61	11	50.00	49.72	99.44	1.68
6	100.00	102.13	102.13	1.72	12	100.00	101.3	101.3	3.29

## Data Availability

All data generated from this study are included in this published article and supporting information. Raw data are available from the corresponding author on reasonable request.
